# *Posidonia oceanica* as a Renewable Lignocellulosic Biomass for the Synthesis of Cellulose Acetate and Glycidyl Methacrylate Grafted Cellulose

**DOI:** 10.3390/ma6052043

**Published:** 2013-05-15

**Authors:** Alessia Coletti, Antonio Valerio, Elena Vismara

**Affiliations:** Department of Chemistry, Materials and Chemical Engineering “G. Natta”, Politecnico di Milano, via Mancinelli 7, Milan 20131, Italy; E-Mails: alessia.coletti@chem.polimi.it (A.C.); antonio.valerio@chem.polimi.it (A.V.)

**Keywords:** *Posidonia oceanica*, *egagropili*, high grade cellulose, crystallinity index, cellulose acetate, glycidyl methacrylate grafted cellulose, wastewater treatment

## Abstract

High-grade cellulose (97% α-cellulose content) of 48% crystallinity index was extracted from the renewable marine biomass waste *Posidonia oceanica* using H_2_O_2_ and organic peracids following an environmentally friendly and chlorine-free process. This cellulose appeared as a new high-grade cellulose of waste origin quite similar to the high-grade cellulose extracted from more noble starting materials like wood and cotton linters. The benefits of α-cellulose recovery from *P. oceanica* were enhanced by its transformation into cellulose acetate ***CA*** and cellulose derivative ***GMA-C***. Fully acetylated ***CA*** was prepared by conventional acetylation method and easily transformed into a transparent film. ***GMA-C*** with a molar substitution (MS) of 0.72 was produced by quenching Fenton’s reagent (H_2_O_2_/FeSO_4_) generated cellulose radicals with GMA. GMA grafting endowed high-grade cellulose from *Posidonia* with adsorption capability. ***GMA-C*** removes β-naphthol from water with an efficiency of 47%, as measured by UV-Vis spectroscopy. After hydrolysis of the glycidyl group to glycerol group, the modified ***GMA-C*** was able to remove *p*-nitrophenol from water with an efficiency of 92%, as measured by UV-Vis spectroscopy. α-cellulose and ***GMA-Cs*** from *Posidonia* waste can be considered as new materials of potential industrial and environmental interest.

## 1. Introduction

In the Mediterranean Sea *Posidonia oceanica* is the dominant seagrass covering about 50,000 km^2^ of coastal sandy areas [1]. The distribution of *P. Oceanica* leaves is strictly linked to underwater light penetration [2]. Its fibrous residues are ball-shaped dry materials, which are called *egagropili* and are found in large amounts along the Mediterranean coast. Banquettes of * P. oceanica* are removed to clean touristic beaches; for example, 114 km of Sardinian coasts in 2004 produced a total amount of about 106,000 m^3^ of banquette, with a range between 0.5 and 1725 m^3^ of sediment subtracted from each beach [3].

The *egagropili* chemical composition has been widely investigated. *Egagropili* has been first submitted to extractive procedures. The quantity of extracted substances in different liquids (water, 1% sodium hydroxide solution and ethanol-toluene) was determined to be around the 35%. The dry residue was made of holocellulose, lignin and a small amount of ash. Holocellulose is the total carbohydrate component and has been calculated to be 61.8% and cellulose contributes 40% [4]. This result makes *egagropili* a renewable cellulose source. The extraction of cellulose from *egagropili* can turn *egagropili* into a resource that provides economic benefits and positive feedback from an environmental point of view [5]. Cellulose extracted from *P. oceanica* has been proposed as a starting material for carboxymethylcellulose [5,6] and for sodium cellulose carboxymethylate [7], which are used as absorption materials.

*Egagropili* does not differ from lignocellulosic materials whose composition is made by carbohydrates, lignin, extractives and minerals [8]. The three main components, native cellulose, hemicellulose and lignin, are always present in different compositions depending on the natural origin of the material. Native cellulose extraction from natural lignocellulosic materials utilises the relative chemical stability of cellulose, which is associated with a total insolubility in water and in most organic solvents [9]. Hydrolysed hemicellulose and cellulose oligomers are separated from cellulose using their specific solubility properties. Lignin is a fairly stable polymer network that acts as a glue to hold the lignocellulosic matrix together. Delignification processes depolymerise and degrade lignin clearing the cellulose chains. After delignification, the soluble lignin degradation products can be separated from the residual insoluble cellulose [10].

In this work, *P. oceanica* was considered as a valuable source of α-cellulose, which can be transformed into cellulose acetate (***CA***) and glycidyl methacrylate grafted cellulose (***GMA-C***). α-cellulose indicates cellulose that is very pure [10]. An environmentally friendly and chlorine-free process was applied for the extraction of α-cellulose from *P. oceanica*. This process has been used to extract α-cellulose from industrial and agricultural waste by-products, such as short flax fibres, ears of corn and wheat straw. It involves a two-step oxidative method, in which the delignification and solubilisation of the components different from cellulose were realised using acetic acid, formic acid and hydrogen peroxide [11]. To the best of our knowledge, the available literature is focused towards the cellulose extraction from *Posidonia*, but at present there are no reports of the extraction of high-grade cellulose. To investigate its quality we studied its acetylation as a novel reaction with cellulose extracted from *Posidonia.*

Cellulose acetate (***CA***) is an artificial fibre whose market is becoming larger and more diversified because ***CA*** is used to obtain textiles, cigarette filters, thermoplastics in combination with plasticisers, LCD flat screens and membranes [12]. ***CA*** has even found application in the automotive industry [13]. Cellulose acetate is usually obtained from high-quality cellulose, such as specially cleaned cotton linters and wood pulps, that have an α-cellulose content of higher than 95% [10,12]. Both cotton linters and wood pulps are expensive starting materials. While cotton linters are fairly pure cellulose, the yield of high-purity cellulose from wood is approximately 50% because wood fibres are composed of cellulose, hemicellulose and lignin, which have to be removed [10]. Therefore, the use of a renewable biomass as an alternative source of cellulose for the production of ***CA*** would be an achievement from both economic and environmental points of view [14]. The achievement of obtaining ***CA*** from *egagropili* perfectly agrees with the strategy of finding sources alternative to wood and cotton linters for ***CA*** production whose importance is increasing day by day [15,16].

Another target of this work was to extend to α-cellulose a glycidyl methacrylate (GMA) grafting approach we worked up on cellulose fibres like cotton and flax.

GMA grafted cellulose fibres have been prepared by an innovative procedure which inserts GMA branches onto a cellulose surface using two steps [17]:

(i) Cellulose activation, which involves radical activation via physical (electron beam and plasma) or chemical (Fenton’s reaction) processes;
(ii) Cellulose grafting, which involves radical quenching via GMA grafting to produce ***GMA-C***.

GMA grafted cellulose fibres have been designed to impart to cellulosic materials specific properties of absorption and release of molecules, e.g., for removing aromatic pollutants from wastewater [18].

GMA grafted cellulose fibres, in which the cellulose is common cotton gauze, have been effective for the adsorption of β-naphthol, yielding GMA appendages specific for non-polar interactions with β-naphthol [18]. GMA-gauzes have been further modified by hydrolysing the glycidyl groups into glycerols. Glycerol appendages appeared to improve the capability of GMA-gauze to adsorb polar nitrophenols as discussed in detail in [18]. Cotton gauzes did not adsorb at all either β-naphthol or nitrophenols confirming the active roles of GMA appendages in capturing them from water.

The aim of obtaining ***GMA-Cs*** from *egagropili* was to have new materials where the GMA grafting adds new adsorption properties to α-cellulose.

## 2. Results and Discussion

### 2.1. Extraction and Characterisation of α-Cellulose

**Figure 1:**
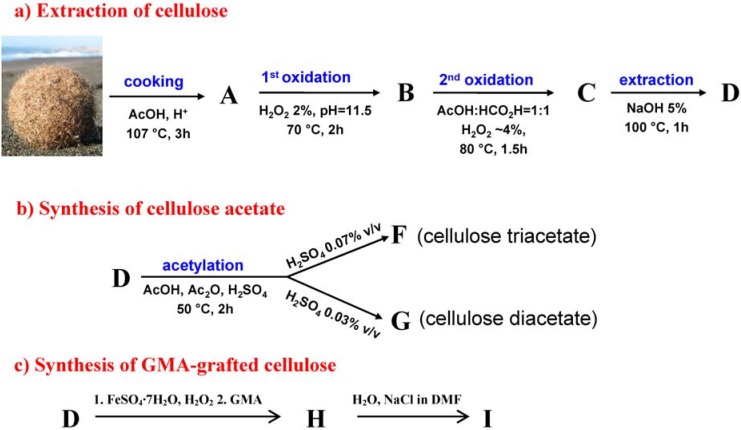
(**a**) Scheme of the extraction of cellulose from the *egagropili* of *P. oceanica*; (**b**) Synthesis scheme of cellulose acetate (***CA***) obtained using the cellulose that was extracted from *P. oceanica*; (**c**) Synthesis scheme of cellulose acetate (***GMA-C***) obtained using the cellulose that was extracted from *P. oceanica*.

The starting *egagropili* was heated in the presence of acetic acid to obtain material **A** according to the acetosolv process [18]. Cellulose was extracted from **A** using the two-step oxidative approach in Figure 1a that avoids the use of chlorinated substances, in accordance with the recent green chemistry rules [19].

**A** was subjected to the first oxidation step, in which hydrogen peroxide in an aqueous base solution causes partial delignification of the starting material to form **B**. Hydrogen peroxide has been used as a replacement bleaching agent for chlorine containing chemicals like ClO_2_, Cl_2_ and NaOCl by the textile industries for many years [20]. During the second oxidative step, the *in situ* formation of performic acid and peracetic acid causes the further depolymerisation of the lignin contained in **B** to obtain material **C**. The last step is an extraction with soda, which enables the dissolution of all of the modified oligosaccharides and monosaccharides that are produced in the previous steps.

The residual material **D** appeared as a white powder. The α-cellulose content of **D** (%) was measured and was found to be 97%. As the α-cellulose content (%) is the parameter usually considered to evaluate the purity of cellulose, we can argue that **D** is pure cellulose. The overall yield of **D** recovering from *egagropili* was calculated around 20%, the yield of the single step being detailed in the experimental part.

**Figure 2:**
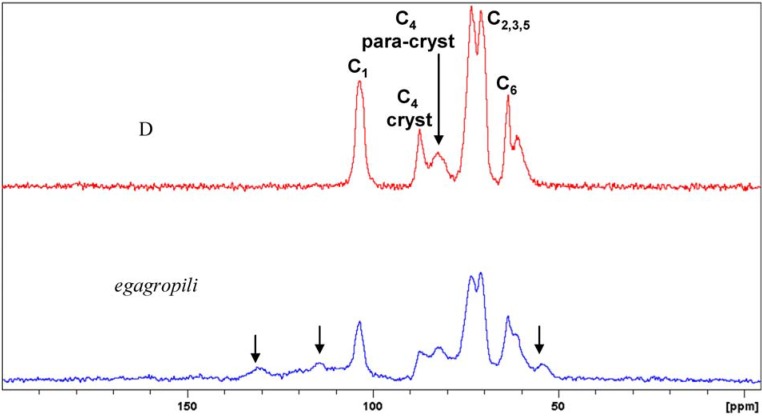
Comparison between the ^13^C CP-MAS NMR spectra of *P. oceanica* (blue) *egagropili* and cellulose extracted from *P. oceanica* (red) sample **D** with the corresponding signal attributions. The arrows in the blue spectrum indicate signals that belong to lignin.

In Figure 2, the solid state Cross Polarization Magic Angle Spinning (^13^C CP MAS) NMR spectrum of **D** was compared with the raw starting material spectrum, where lignin signals are highlighted by arrows in the Figure. The two spectra visualise not only the trivial difference in composition but also the morphology before and after the extraction procedure. The signals due to the cellulose components between 60 and 90 ppm show the enrichment of the crystalline component in sample **D**. ^13^C CP MAS NMR is widely used to investigate native celluloses [21]. CP MAS NMR emphasizes the crystalline and paracrystalline components [22]. Cellulose consists of crystalline and non-crystalline phases in different ratios, depending on the natural source. The crystallinity index (Cr.I.) of cellulose can be calculated from the ^13^C CP MAS NMR spectra by integrating the C_4_ signals, which resonate at 85–90 ppm (range a) for crystalline cellulose and at 79–85 ppm (range b) for non-crystalline cellulose, as shown in Figure 2. The Cr.I. of **D** was evaluated as in Equation (1) [23], giving a Cr.I. value of 48%:

(1) $$Cr.I.=\frac{a}{\left(a+b\right)}\times 100$$

In the crystalline phase, the cellulose fibres are ordered by inter- and intramolecular hydrogen bonds, while in the non-crystalline phase, the network does not have an organised structure [24]. The differences in the structures determine the different accessibilities of the hydroxyl groups during the synthesis of cellulose acetate. The amorphous part consists of random orientation of cellulose chains. As the accessibility for reagents is much better in the amorphous phases, Cr.I. is a crucial parameter for cellulose functionalisation [25].

α-Cellulose content and Cr.I. described **D** in term of purity and morphology, respectively. Bleached sulphite wood pulp from Borregaard company (WPB) used to produce cellulose acetates was chosen as our target. WPB α-cellulose content was 95.2% *versus* 97% of **D** and Cr.I. was 53.9%, *versus* 48% of **D**. Thus **D** can be reasonably assessed a starting material suitable for cellulose acetate production.

### 2.2. Synthesis and Characterisation of Cellulose Acetate (**CA**)

**Figure 3:**
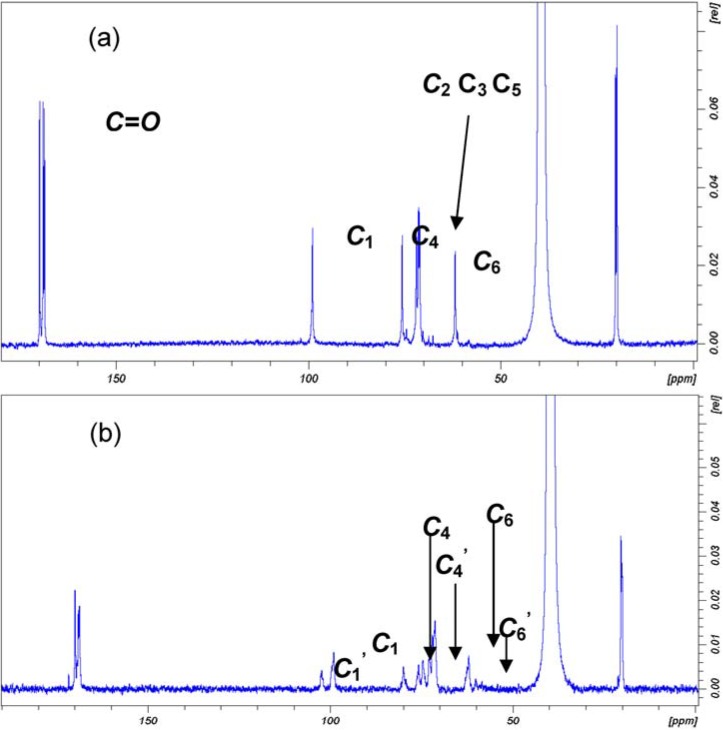
Comparison between the ^13^C NMR spectra of cellulose triacetate **F** (**a**) and cellulose diacetate **G**; (**b**) in DMSO-d6 in the presence of Chromium(III) acetylacetonate (*T* = 323 K). Attribution of the signals is given for compound **F** (**a**); signal used for calculation of DS are evidenced in (**b**), where the signals of unsubstituted positions are indicated as C_n_′.

**Figure 4:**
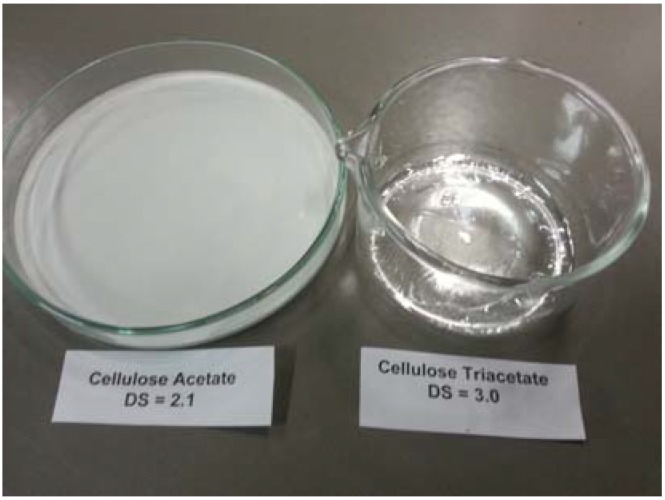
Evaporation of dichloromethane **F** and **G** solutions. The **F** transparent film.

Cellulose acetate application and performance have been exhaustively described, including their capacity for film formation [26]. The synthetic pathway used to obtain cellulose acetates **F** and **G** from **D** is summarised in Figure 1b. Acetic anhydride was used as an acetylating agent in an acidic medium. **D** was fully acetylated affording an almost quantitative yield (93%) of pure cellulose triacetate **F**, as can be observed in the ^13^C NMR spectrum in Figure 3 and substitution degree (DS) calculation in Table 1. DS that describes the amount of acetyl groups introduced by reaction of the cellulose hydroxyl groups was determined using the ^13^C NMR technique as summarised in the experimental section. **F** was completely soluble in dichloromethane, and its transparent film was obtained by evaporating dichloromethane from a concentrated solution in the absence of any plasticizer, see Figure 4.

**Table 1:** Determination of the DS of ***CA*** **F** and **G** from the ^13^C NMR measurements in DMSO-d6 in the presence of Chromium(III) acetylacetonate (*T* = 323 K).

Entry	$DS2=1-\frac{\left({\text{C}}_{1}-\text{OH}\right)}{\left({\text{C}}_{1}-\text{OAc}+{\text{C}}_{1}-\text{OH}\right)}$	$DS3=1-\frac{\left({\text{C}}_{4}-\text{OH}\right)}{\left({\text{C}}_{1}-\text{OAc}+{\text{C}}_{1}-\text{OH}\right)}$	$DS6=\frac{\left({\text{C}}_{6}-\text{OAc}\right)}{\left({\text{C}}_{6}-\text{OAc}+{\text{C}}_{6}-\text{OH}\right)}$	DS_tot_
F	3	3	3	3
G	0.71	0.64	0.85	2.2

The formation of partially substituted cellulose acetate **G** in 73% yield was possible by slight change in the reaction conditions, *i.e.*, by using a smaller amount of catalytic acid, see its ^13^C NMR spectrum in Figure 3 and substitution degree (DS) calculation in Table 1. **G** was slightly soluble in dichloromethane. Transparent film formation was not observed by evaporating its dichloromethane suspension, see Figure 4.

### 2.3. Synthesis and Characterisation of GMA Grafted Cellulose (**GMA-C**)

Cellulose can be functionalised by grafting GMA to polymeric chains to obtain the modified cellulose ***GMA-Cs*** as summarised in Figure 1c.

**Figure 5:**
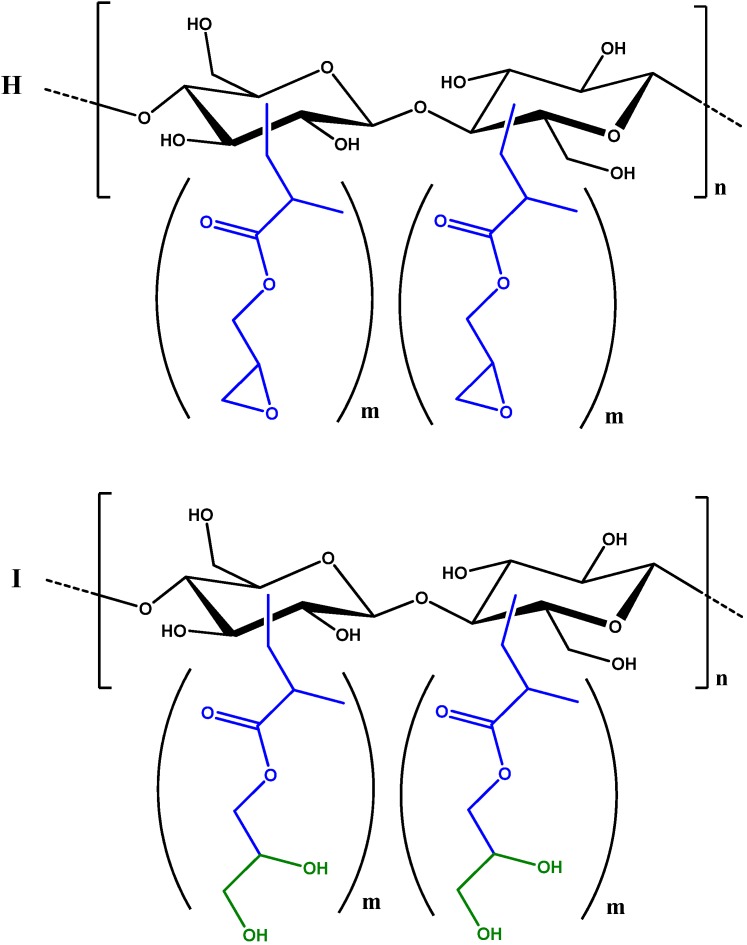
***GMA-C*** (sample **H**) and modified sample **I** by hydrolytic epoxide ring opening.

Based on previous results, **D** was functionalised with GMA via Fenton’s reaction to produce sample **H**, which was successively hydrolysed to obtain glycerol branched **I** (Figure 5).

**Figure 6:**
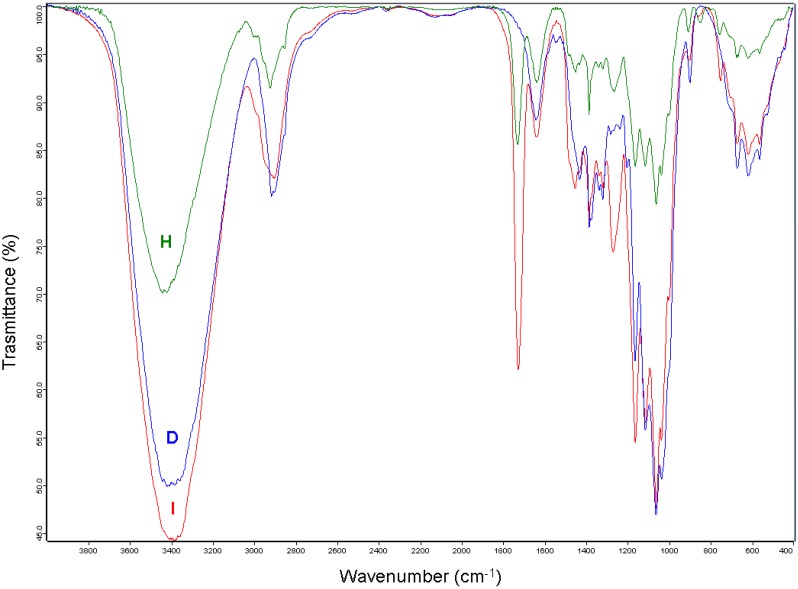
FT-IR spectra of cellulose **D** and cellulose derivatives **H** and **I** in a KBr pellet.

Figure 6 shows the FT-IR spectra profiles of cellulose **D** and of the ***GMA-C*** samples **H** and **I**. FT-IR spectroscopy is a common technique used to investigate the structural changes of cellulosic material upon chemical reactions. The FT-IR spectrum of cellulose **D** presents the bands typical of cellulose: 3400 cm^−1^ (O–H stretching); 2910 cm^−1^ (C–H stretching); 1640 cm^−1^ (absorbed water); 1430 cm^−1^ (CH_2_ bending); 1380 cm^−1^ (O–H bending); 1320 cm^−1^ (C–C and C–O vibrations); 1220–920 cm^−1^ (C–O asymmetric bridge stretching and C–O–C pyranose ring skeletal vibrations); 895 cm^−1^ (β-glycosidic linkage) [23]. The comparison between the FT-IR spectra of cellulose **D** and of cellulose derivative **H** demonstrates the functionalisation of the glucose units, since two new signals appear at 1730 cm^−1^ and at 1270 cm^−1^ due to the C=O stretching and to the –C–O– stretching of the ester functional group introduced by grafting GMA, together with the signal at 848 cm^−1^ due to C–O–C epoxide ring stretching.

103 mg of **H** was obtained in 50 yield%. **H** molar substitution degree (MS_FT-IR_ = 0.72), were calculated from the FT-IR spectrum as detailed in the experimental section.

**Figure 7:**
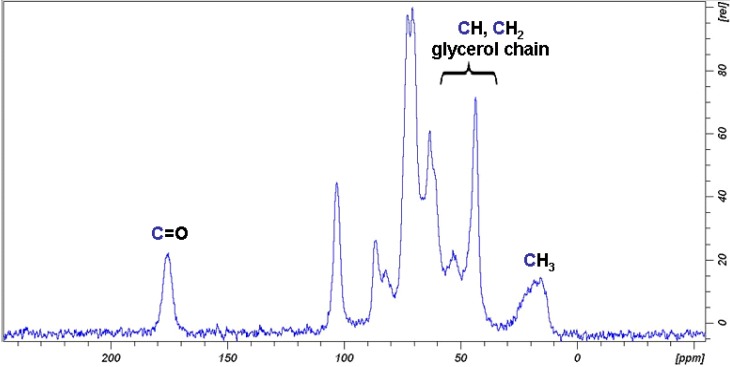
^13^C CP MAS NMR spectrum of sample **I**.

**I** was obtained from **H** through a ring opening reaction in 78 yield% and it was characterised by both FT-IR and by ^13^C CP MAS NMR. The FT-IR spectrum is similar to that of the starting material **H**, the only difference being the disappearance of the epoxide signal at 848 cm^−1^ (Figure 6). The signals of the glycerol group formed after the ring opening reaction are in the range of 3000–3600 cm^−1^, where cellulose hydroxyl groups signals are prominent. The ^13^C CP MAS NMR spectra of **I**, shown in Figure 7, is consistent with that reported for glycerol branched ***GMA-C*** obtained from cotton gauzes as cellulosic material [18]. Comparing the spectrum with that of the starting material **D** (Figure 2), it is evident that the main difference is the appearance of a series of new signals due to the introduction of the glycerol branching. The signal centred at 175 ppm corresponds to the presence of a carbonyl group, in agreement with FT-IR spectrum; the signals appearing in the range of 10–60 ppm are due to the presence of the methacrylate CH_3_ groups.

### 2.4. Absorption of β-naphthol and of p-nitrophenol

**Figure 8:**
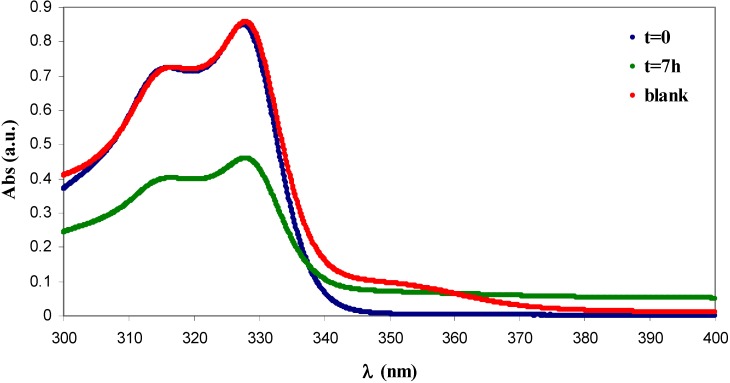
UV-Vis spectra of β-naphthol (BN) aqueous solutions. *Blue*: [BN] = 5 × 10^−4^ M before the addition of cellulose. *Green*: 7 h after the addition of **H**. *Red*: 24 h after the addition of α-cellulose **D**.

**Figure 9:**
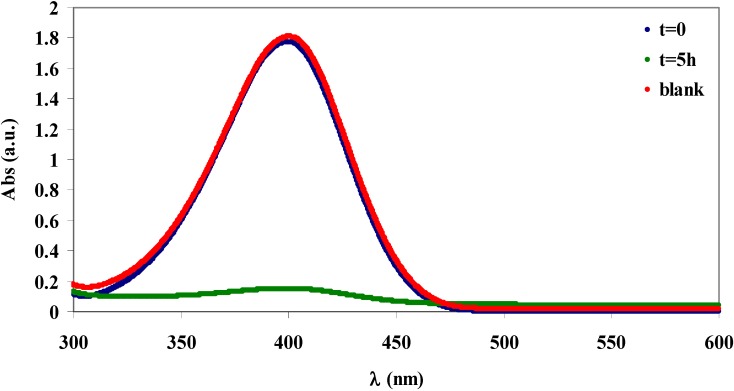
UV-Vis spectra of NP aqueous solutions. *Blue*: [NP] = 1 × 10^−4^ M before the addition of cellulose. *Green*: 5 h after the addition of **I**. *Red*: 5 h after the addition of α-cellulose **D**.

Cotton cellulose functionalised with GMA was applied for the adsorption of aromatic pollutants from wastewaters [18]. **H** and **I** were studied for the same application with the aim of providing GMA grafted α-cellulose materials which can appreciate α-cellulose use in novel directions. Some aromatic pullatants, β-naphthol (BN) and *p*-nitrophenol (NP), (water solution, at the same concentration) were tested with DS ***GMA-C***, 0.72 *versus* 0.65 of GMA grafted cotton gauze. The saturation of GMA grafted cotton gauze with BN and NP occurred after a contact time of seven hours, so the same contact time hold over for ***GMA-C***. **H** was tested for its capability of adsorption of BN (see Figure 8) while **I** was tested for its capability of adsorption of NP (see Figure 9). To calculate the amount of absorbed BN and NP, the UV-Vis spectra of the corresponding aqueous solutions were recorded. In fact, the depletion of the UV-Vis absorption is quantitatively correlated to the amount of absorbed pollutant according to Equation (2) where *C*_0_ is the initial concentration and *C*_eq_ is the final concentration.

(2) $$\%removal=\frac{C_{0}-C_{eq}}{C_{0}}\times 100$$

**H** was effective in the adsorption of BN. Figure 8 shows the absorption spectra of the resulting aqueous solutions of BN. The initial concentration of β-naphthol was 5.0 × 10^−4^ M. The concentration of BN decreased to 2.7 × 10^−4^ M for 7 h after the addition of **H**, as calculated by evaluating the absorption of the aqueous solution at 328 nm (green line). Therefore, the removal efficiency of **H**, calculated by Equation (2), is 47%, which corresponds to an absorption capacity of 4.7 × 10^−5^ mol/g. This value is comparable to that obtained with GMA grafted cotton gauze, (1.07 × 10^−5^ mol/g) [17].

The same result was obtained after 24 h (not shown), indicating that the equilibrium plateau was already reached after 7 h. The same experiment conducted with **D** did not remove the organic pollutant at all, as shown by the red line in Figure 8, which overlays the absorption spectrum of the initial BN aqueous solution (blue line).

Following the same experimental procedure used for BN, a 1.0 × 10^−4^ M solution of NP was treated with **I** and with **D**. The absorption spectra of the resulting aqueous solutions are shown in Figure 9. Under these experimental conditions, with **I** as the absorption material, NP was almost completely removed from the water with a removal efficiency of 92%, which corresponds to an absorption capacity of 1.9 × 10^−5^ mol/g. In fact, the absorption band of the pollutant centred at 400 nm (blue line) almost totally disappeared after treatment with **I** (green line). This value is comparable to that obtained with analogue GMA grafted cotton gauze, 5.2 × 10^−5^ mol/g [17]. The blank experiment obtained by treating for 5 h an aqueous solution of NP with **D** was superimposable with that of the initial conditions (red line), demonstrating the lack of any absorption capability of **D**.

The affinity of **H** to BN and the affinity of **I** to NP are in complete agreement with the previous results that were obtained for GMA grafted cotton gauze [18]. Same way cotton gauze did not show any affinity and absorption capability towards BN and NP.

## 3. Experimental Section

### 3.1. Materials and Methods

The *egagropili* of *P. Oceanica* that were used in this study as dry material were collected on Sardinian beaches. Before use, they were washed with hot acetone and water to eliminate contaminants and residual sand, oven dried (60 °C, 24 h) and ground. Acetic acid 90%, sulphuric acid 96%, formic acid 85%, acetic anhydride 98%, NaOH 97% and NaHCO_3_ 99.7% were purchased from Carlo Erba Reagenti and used as received. H_2_O_2_ 30% (w/w), KOH > 85%, GMA 97%, FeSO_4_·7H_2_O, β-naphthol 99% and *p*-nitrophenol 99% were purchased from Sigma-Aldrich and used as received.

Solid state ^13^C CP MAS NMR spectra were recorded on a Bruker (Germany) ASX 300 spectrometer operating at 74.47 MHz. Solution ^13^C NMR spectra were recorded with a Bruker (Germany) AMX 400 spectrometer.

The solid phase FT-IR spectra of the powder samples **D**, **H** and **I** were performed with infrared grade KBr on an Bruker Alpha (Germany) spectrometer, with resolution of 4 cm^−1^.

### 3.2. Extraction of Cellulose

The extraction of cellulose from the *egagropili* of *P. oceanica* was obtained using four steps. (i) *Cooking*: First, 5.0 g of *egagropili*, treated as described before, was added to 200 mL of 90% acetic acid, 500 μL of 96% H_2_SO_4_ was added to the suspension as a catalyst and the reaction mixture was heated at 107 °C under stirring for 3 h. The solid was recovered by filtration, washed with distilled water until pH 7 was obtained and dried at room temperature to produce sample **A**. Yield: 64%; (ii) *Oxidation with H_2_O_2_*: 3.2 g of the obtained material **A** was suspended in 160 mL of distilled water, and 11.2 mL of H_2_O_2_ (30% w/w) was added to reach a final concentration of 2%. NaOH, 4M, was added until pH 11.5 was reached. The reaction was heated at 70 °C and vigorously stirred for 2 h. NaOH was added to maintain the alkaline pH. The reaction mixture was left to cool at room temperature and filtered. The solid was washed with distilled water until a neutral pH was reached to obtain sample **B**. Yield: 84%; (iii) *Oxidation with peracids*: 2.7 g of **B** was added to 46 mL of a 1:1 mixture of 96% acetic acid and formic acid. After the addition of 6.8 mL of H_2_O_2_ (30% w/w), the mixture was heated at 80 °C under stirring for 1.5 h. After the mixture was cooled, the solid was recovered by filtration, washed first with 60 mL of a NaHCO_3_ saturated solution and then with distilled water to a neutral pH and dried in air, producing sample **C**. Yield: 58%; (iv) *Extraction with NaOH*: 1.56 g of the solid **C** was suspended in 10 mL of NaOH (5% w/w) at 100 °C under magnetic stirring for 1 h. After it cooled, the reaction mixture was filtered, and the solid was washed to a neutral pH with distilled water and acetone and left to dry in air, obtaining sample **D**. Yield: 64%.

### 3.3. Determination of α-Cellulose Content

The α-cellulose content was determined following the procedure described by Rodriguez Filho *et al.* [27] and Veira *et al.* [28]. Sample **D**, 0.6 g, was suspended in 20 mL of a 5% KOH (w/w) solution and stirred at 25 °C for 2 h. under a nitrogen atmosphere. The mixture was filtered on a sintered crucible and washed first with 10 mL of a 5% KOH (w/w) solution and then with 20 mL of distilled water. Using the same procedure, the obtained fibrous solid was mixed with 20 mL of a 24% KOH (w/w) solution and stirred for 2 h at 25 °C. The reaction mixture was filtered, and the solid was washed with 5 mL of 24% KOH (w/w), 5 mL of a 10% acetic acid solution and distilled water to obtain a neutral pH. The solid was washed with 20 mL of acetone and dried at 105 °C overnight. Yield: 97%.

### 3.4. Solid State ^13^C CP MAS NMR

^13^C CP MAS NMR spectra were recorded following a previously published procedure [11] with slight modifications. A ^1^H 90° pulse length of 3.2 μs, a repetition time of 8 s, a contact time of 1.2 ms and a spin rate of 5000 Hz were used. The compounds were placed in a zirconium rotor, which was 7 mm in diameter and 21 mm high. The chemical shifts were calibrated relative to tetramethylsilane with benzene as secondary reference.

### 3.5. Synthesis of Cellulose Acetate

Sample **D**, 0.455 g, was swelled in 15 mL of acetic acid at 50 °C, and 2 mL of acetic anhydride (Ac_2_O) and 100 μL of 96% sulphuric acid were added under stirring (1 h). A second portion of Ac_2_O (2 mL) was added, and the reaction was run for 1 more h at 50 °C. The obtained homogeneous solution was cooled at room temperature and poured into 200 mL of water at 0 °C. The mixture was left at 4 °C overnight. The obtained solid was recovered by filtration, washed first with an NaHCO_3_ saturated solution and then with distilled water and air dried to obtain 0.75 g of sample **F**. Yield: 93%.

Sample **G** was prepared starting from 1.0 g of **D**. **D** was swelled in acetic acid (60 mL) under stirring at 50 °C, and 2 mL of Ac_2_O and 200 μL of H_2_SO_4_ were then added. The reaction was stirred at 50 °C for 1 h. After the addition of other 2 mL of Ac_2_O, the reaction was further run for 2 h under stirring at 50 °C. The homogeneous solution was left to cool at room temperature, poured into 600 mL of a saturated NaHCO_3_ solution at 0 °C and stored at 4 °C overnight. The mixture was filtered, and the obtained solid was washed with distilled water and air dried to obtain 1.14 g of sample **G**. Yield: 73%.

Samples **F** and **G**, 146 mg, were separately dissolved in 7 mL of dichloromethane at room temperature and stirred for 5 min. The solutions were transferred into petri dishes to allow the solvent to evaporate in air. After 15 min, the formation of a transparent film from **F** was observed over the glass surface.

### 3.6. Solution ^13^C NMR

Sample **F**, 51 mg, and Chromium(III) acetylacetonate, 35 mg, was dissolved in 2.5 mL of DMSO-d6 at 323 K for 3 h in an ultrasound bath. A sample temperature of 323 K, SN = 16 k, an acquisition time AQ of 600 ms, a spectral window of 260 ppm (SW) and a recycle time D1 = 5 s (pulse program zgig) were used. The NOE was absent. The use of two expedients enables the use of the signal ratios of the equivalent atoms of ^13^C NMR for quantitative analysis. To homogenise the relaxation times of all of the ***CA*** atoms, the NMR spectra of **F** and **G** were obtained in the presence of Chromium(III) acetylacetonate; moreover, to reduce the Nuclear Overhauser Effect (NOE), the decoupling power was gated off during the pulse sequence. The DS was calculated separately for the primary and secondary carbon atoms C_2_, C_3_ and C_6_. In the ^13^C NMR spectrum of the cellulose chains, the introduction of acetyl groups determined the shift of all the signals. This effect is particularly evident for the C_1_, C_4_ and C_6_ atoms, which exhibit signals that are due to substituted (C–OAc) and unsubstituted (C–OH) positions. Therefore, DS6 was calculated using the integrals of the C_6_ signals, while DS2 and DS3 were calculated using the signals that belong to C_1_ and C_4_, which are the atoms adjacent to the carbons C_2_ and C_3_, respectively. The equations that were used for each carbon atom are given in Table 1.

### 3.7. Functionalisation of Cellulose for the Adsorption of Organic Aromatic Pollutants

In a one-neck, round-bottomed flask, 125 mg of cellulose **D** was swelled in 37.5 mL of water at 80 °C under stirring for 1 h. Then, 1 mL of a 0.05 M freshly prepared solution of FeSO_4_·7H_2_O in water and 6.5 mL of 30% H_2_O_2_ (w/w) were added, and the mixture was stirred for 30 min. Finally, 0.5 mL of GMA was added under vigorous stirring, and the reaction was run for 15 min. During all of the three reaction steps, the temperature was 80 °C.

Raw ***GMA-C*** was recovered by vacuum filtration through a Buchner funnel and washed with distilled water (approximately 10 mL) and acetone (approximately 30 mL). To further purify the sample to remove the GMA homopolymer, which was produced as a side reaction, cellulose was swollen in a round-bottomed flask with 75 mL of acetone under reflux for 2.5 h and recovered by filtration. The sample was dried in an oven at 60 °C overnight to obtain 103 mg of **H**. Yield: 50%.

To open the epoxide ring, sample **H** was reacted with water. **H**, 75 mg, was swelled in a round-bottomed flask with 3 mL of DMF at 80 °C for 2 h. Then, 3 mL of 1 M aqueous NaCl were added, and the mixture was stirred for 22 h at 80 °C. The solid was recovered by filtration through a Buchner funnel, washed with 50 mL of water and 25 mL of acetone and dried in an oven at 60 °C for two hours to obtain 59 mg of sample **I**. Yield: 78%.

### 3.8. Quantification of GMA Grafting by FT-IR Spectroscopy

As demonstrated in a previous work, the ester stretching characteristic signal of the grafted GMA at 1730 cm^−1^ can also be used to quantify the functionalisation [18]. The area of the ester band was normalised in reference to another characteristic cellulose band (780–465 cm^−1^), as shown by Equation (3). The numerical values MS_FT-IR_ that were obtained from Equation (3) can be used for absolute quantification because they correlate well with the “molar substitution ratio”, MS_w_, which can be determined from gravimetric measurements and from Equation (4). MS_w_ is the average molar ratio between the grafted GMA units and the anhydrous D-glucose units.

(3) $${\text{MS}}_{\text{FT}-\text{IR}}={\text{area}}_{\text{N(ester)}}=\frac{{\text{area}}_{\text{ester}}(\text{manual}\;\text{band}\;\text{integration})}{{\text{area}}_{\text{cellulose}}(\text{range}\;780-465\text{c}{\text{m}}^{-1}\;\text{integration})}$$

(4) $$\text{M}{\text{S}}_{w}=\frac{\left(m-m_{0}\right)\times 162}{m_{0}\times 142}$$

In Equation (4), *m* and *m*_0_ (g) are the final mass and the initial mass of the samples, respectively; 162 is the molar mass of d-glucose and 142 is the molar mass of GMA.

The good correlation between MS_FT-IR_ and MS_w_ demonstrates that the molar extinction coefficients of IR absorption bands of the GMA ester stretching and those of the chosen characteristic cellulose band are similar.

### 3.9. Absorption Experiments

A stock solution of BN was prepared by dissolving 18 mg of β-naphthol in 50 mL of water in a volumetric flask and shaking the solution overnight at 28 °C. **D** and **H**, 20 mg, were separately immersed in 4 mL of a 5 × 10^−4^ M BN aqueous solution, which was prepared by diluting the stock solution. The mixtures were shaken for 24 h at 100 rpm in a thermostatic bath at 25 °C. The adsorption of BN was monitored using the UV-Vis measurements, from 300 to 700 nm, of the aqueous solutions, which were filtered and measured after 5 and 24 h and compared with an untreated solution (time = 0). The concentration of BN was calculated using the absorbance of β-naphthol at 328 nm (molar absorptivity of 1734 M^−1^ cm^−1^).

To determine the absorption of the NP experiments, a 2.5 × 10^−3^ M stock solution was prepared by dissolving 17.4 mg of *p*-nitrophenol in 50 mL of approximately 1 × 10^−3^ M NaOH and shaking the solution overnight at 20 °C. The stock solution, 400 μL, was diluted with 1 × 10^−3^ M NaOH until a total volume of 10 mL was obtained. The final concentration of NP in the diluted solution was 1.0 × 10^−4^ M, and the pH value was 10.5. 20 mg of celluloses **D** and **I** were separately immersed in 4 mL of 1.0 × 10^−4^ M NP and shaken at 25 °C for 24 h. The adsorption of NP was monitored using the UV-Vis measurement of the aqueous solutions, which were filtered and measured after 5 h and 24 h. The concentration of NP was evaluated using the absorption value at 400 nm (molar absorptivity of 18,380 M^−1^ cm^−1^).

## 4. Conclusions

Recovering a noble component from waste instead of destroying it was the first challenge of this study. The valorisation of a marine biomass from *P. oceanica* was achieved by recovering high-quality cellulose, a strategic industrial cellulose material. It was further transformed into cellulose triacetate ***CA*** and into ***GMA-Cs*** materials. These achievements offer real industrial perspectives and work-up possibilities for *P. oceanica*. ***GMA-Cs*** were found to be effective in capturing β-naphthol and *p*-nitrophenol, which are important toxic agents in wastewater. The source and the physical aspect of the ***GMA-Cs*** make them excellent cellulose-base materials to develop in the field of filtration water technology.

## References

[1] Fornes A., Basterretxea G., Orfila A., Jordi A., Alvarez A., Tintore J. (2006). Mapping Posidonia oceanica from IKONOS. ISPRS J. Photogramm..

[2] Dalla Via J., Sturmbauer C., Schönweger G., Sötz E., Mathekowitsch S., Stifter M., Rieger R. (1998). Light gradients and meadow structure in Posidonia oceanica: Ecomorphological and functional correlates. Mar. Ecol. Progr. Ser..

[3] De Falco G., Simeone S., Baroli M. (2008). Management of beach-cast posidonia oceanica seagrass on the island of sardinia (Italy, Western Mediterranean). J. Coast. Res..

[4] Khiari R., Mhenni M.F., Belgacem M.N., Mauret E. (2010). Chemical composition and pulping of date palm rachis and Posidonia oceanic—A comparison with other wood and non-wood fibre sources. Bioresour. Technol..

[5] Khiari R., Mhenni M.F., Belgacem M.N., Mauret E. (2011). Valorisation of vegetal wastes as a source of cellulose and cellulose derivatives. J. Polym. Environ..

[6] Aguir C., Mhenni M.F. (2006). Experimental study on carboxymethylation of cellulose extracted from *Posidonia oceanica*. J. Appl. Polym. Sci..

[7] Khiari R., Ferchichi I., Mhenni M.F. (2010). Study of liquids absorption and retention capacities of new cellulosic materials and sodium cellulose carboxylmethylate prepared from posidonia. Fibre Polym..

[8] Keijsers E.R.P., Yılmaz G., van Dam J.E.G. (2013). The cellulose resource matrix. Carbohydr. Polym..

[9] Liebert T.F., Heinze T.J., Edgar K.J. (2010). Cellulose Solvents: For Analysis, Shaping and Chemical Modification.

[10] Saka S., Matsumura H. (2004). Wood pulp manifacturing and quality characteristics. Macromol. Symp..

[11] Vismara E., Gastaldi G., Valerio A., Bertini S., Cosentino C., Eisele G. (2009). Alpha cellulose from industrial and agricultural renewable sources like short flax fibres, ears of corn and wheat-straw and its transformation into cellulose acetates. J. Mater. Chem..

[12] Rustemeyer P. (2004). History of CA and evaluation of the markets. Macromol. Symp..

[13] Mohanty A.K., Misra M., Drzal L.T. (2002). Sustainable bio-composites from renewable resources: Opportunities and challenges in the green materials world. J. Polym. Environ..

[14] Saka S. How Raw Materials Influence the Quality of Cellulose Diacetate. Abstracts of Papers. Proceedings of the 229th ACS National Meeting.

[15] Mondal M.I.H., Alam A.B.M.F. (2013). Utilization of cellulosic wastes in textile and garment industries: 2. Synthesis and characterisation of cellulose acetate from knitted rag. J. Polym. Environ..

[16] He J., Zhang M., Cui S., Wang S.-Y. (2009). High-quality cellulose triacetate prepared from bamboo dissolving pulp. J. Appl. Polym. Sci..

[17] Torri G., Vismara E., Alberti A., Bertini S., Ciardelli G., Gastaldi G., Nesti S. (2010). Free-Radical Functionalised Polysaccharides. US Patent.

[18] Vismara E., Melone L., Gastaldi G., Cosentino C., Torri G. (2009). Surface functionalisation of cotton cellulose with glycidyl methacrylate and its application for the absorption of aromatic pollutants from wastewaters. J. Hazard. Mater..

[19] Anastas P., Eghbali N. (2010). Green chemistry: Principles and practice. Chem. Soc. Rev..

[20] Zeronian S.H., Inglesby M.K. (1995). Bleaching of cellulose by hydrogen peroxide. Cellulose.

[21] Yannoni C.S. (1982). High-resolution NMR in solids: The CPMAS experiment. Acc. Chem. Res..

[22] Atalla R.H., VanderHart D.L. (1999). The role of solid state ^13^C NMR spectroscopy in studies of the nature of native celluloses. Solid State Nucl. Magn. Reson..

[23] Sun J.X., Sun X.F., Zhao H., Suna R.C. (2004). Isolation and characterisation of cellulose from sugarcane bagasse. Polym. Degrad. Stable.

[24] Focher B., Palma M.T., Canetti M., Torri G., Cosentino C., Gastaldi G. (2001). Structural differences between non-wood plant cellulose: Evidence from solid state NMR, vibrational spectroscopy and X-ray diffractometry. Ind. Crops Prod..

[25] Steinmeier H. (2004). Chemistry of cellulose acetylation. Macromol. Symp..

[26] Edgar K.J., Buchanan C.M., Debenham J.S., Rundquist P.A., Seiler B.D., Shelton M.C., Tindall D. (2001). Advances in cellulose ester performance and application. Prog. Polym. Sci..

[27] Rodrigues Filho G., Monteiro D.S., Meireles C.S., de Assunção R.M.N., Cerqueira D.A., Silva Barud H., Ribeiro S.J.L., Massadeq Y. (2008). Synthesis and characterisation of cellulose acetate produced from recycled newspaper. Carbohydr. Polym..

[28] Viera R.G.P., Filho G.R., de Assunção R.M.N., Meireles C.S., Vieira J., de Oliveira G.S. (2007). Synthesis and characterisation of methylcellulose from sugar cane bagasse cellulose. Carbohydr. Polym..

[29] Miyamoto T., Sato Y., Shibata T., Inagaki H., Tanahashi M. (1984). ^13^C nuclear magnetic resonance studies of cellulose acetate. J. Polym. Sci. Polym. Chem. Ed..

